# Dosimetric factors associated with long-term patient-reported outcomes after definitive radiotherapy of patients with head and neck cancer

**DOI:** 10.1186/s13014-019-1429-3

**Published:** 2019-12-09

**Authors:** Toyokazu Hayakawa, Shogo Kawakami, Itaru Soda, Takuro Kainuma, Marika Nozawa, Akane Sekiguchi, Shunsuke Miyamoto, Taku Yamashita, Hiromichi Ishiyama

**Affiliations:** 1 Department of Radiation Oncology, Kitasato University School of Medicine, 1-15-1 Kitasato, Minamiku, Sagamihara Japan; 2 Department of Otorhinolaryngology and Head and Neck Surgery, Kitasato University School of Medicine, 1-15-1 Kitasato, Minamiku, Sagamihara Japan

**Keywords:** Head & neck cancer, Radiotherapy, Health-related quality of life, Pharyngeal constrictor muscle, Parotid gland

## Abstract

**Background:**

The aim of this study was to explore the relationships between dosimetric parameters of organs at risk and patient-reported outcomes (PRO) after radiotherapy of patients with head and neck cancer.

**Methods:**

PRO data of 53 patients with head and neck cancer treated with radiotherapy were prospectively collected. These data concerned health-related quality of life (HRQOL) and were collected using the European Organization for Research and Treatment of Cancer Quality of Life Questionnaire (QLQ-C30) and head and neck cancer module (QLQ-H&N35). Patients were divided into “severe-deterioration” and “mild-deterioration” groups on the basis of degree of deterioration HRQOL > 6 months after completing treatment. The relationships between HRQOL deteriorations and patient-related or dosimetry-related factors were evaluated. *P* < 0.0013 according to Bonferroni correction was considered to denote statistical significance.

**Results:**

Regarding “trouble with social eating (HNSO)” and “coughing (HNCO),” there were significant differences between the severe-deterioration and mild-deterioration groups in mean dosages to the superior pharyngeal constrictor muscle (SPC) (HNSO: 62.5 Gy vs 54.2 Gy; *p* = 0.00029, and HNCO: 61.5 Gy vs 54.1 Gy; *p* = 0.0012) and parotid gland (HNSO: 24.1 Gy vs 20.5 Gy; *p* = 0.000056, and HNCO: 24.2 Gy vs 20.3 Gy; *p* = 0.00043). Regarding “nausea and vomiting,” there was a significant difference between the two groups in the mean dosage to the middle pharyngeal constrictor muscle (MPC: 61.9 Gy vs. 58.4Gy; *P* = 0.00059).

**Conclusions:**

We found that dosages to the SPC and parotid gland were associated with severe deterioration in HRQOL attributable to difficulty in HNSO and HNCO, whereas dosage to the MPC was associated with severe deterioration attributable to nausea and vomiting.

## Background

In the field of radiation oncology, physician-reported toxicity assessed on the basis of National Cancer Institute Common Toxicity Criteria and/or Radiation Therapy Oncology Group criteria had been the standard means of assessing toxicity. Recently, however, the importance of patient-reported outcomes (PRO) has been increasingly recognized because of concerns about large discrepancies between physician-reported and patient-reported toxicity [2].

Although relationships between PRO and dosimetric parameters have been reported by several investigators, these reports were based on assessments at very few time points [4] or cross-sectional analysis [5, 8]. The purpose of this study was, therefore, to explore in more detail the relationships between dosimetric parameters and chronological changes in PRO outcomes.

## Methods

### Patients

**Fig. 1:**
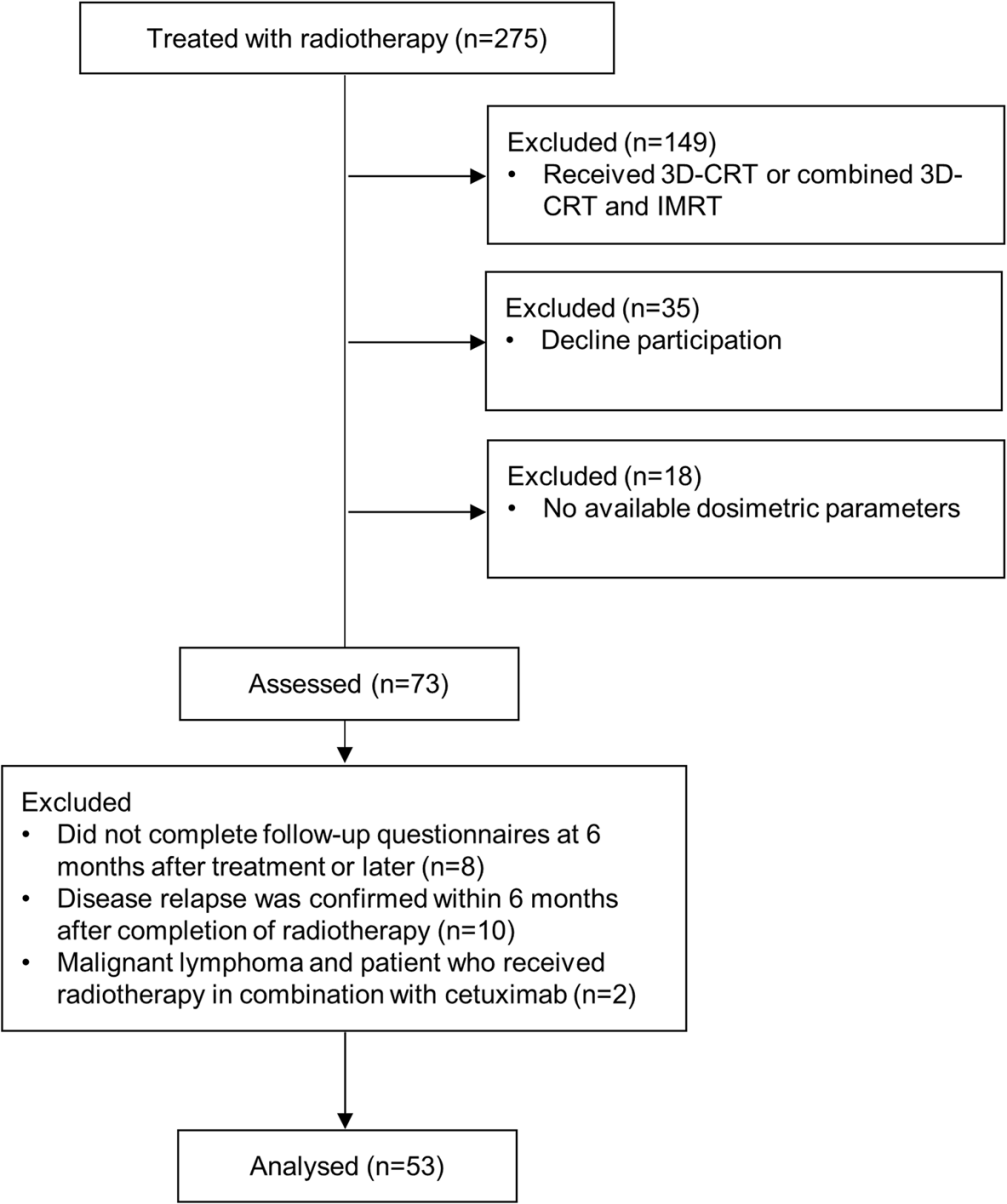
CONSORT diagram of study. Abbreviations: 3D-CRT = three-dimensional conformal radiotherapy; IMRT = intensity-modulated radiotherapy; DVH = dose-volume histogram

Our institutional review board approved this observational study (B12–27). Between August 2014 and March 2017, 275 patients with head and neck cancer attending our department were recruited for prospective health-related quality of life (HRQOL) assessments. All patients gave written informed consent before enrollment. In this analysis, we focused on the relationship between dose to organ at risk (OAR) and deterioration in HRQOL. Therefore, 149 patients treated by three-dimensional conformal radiotherapy (3DCRT) or combined 3DCRT and intensity modulated radiotherapy (IMRT) and 18 patients treated by IMRT were excluded because detailed OAR data were not available. Thirty-five patients refused to participate. A CONSORT diagram is presented in Fig. 1.

Patient and treatment characteristics are summarized in Table 1. The majority of patients were male (91%) and the patients’ median age was 67 years (range, 45 to 89 years). Thirty-nine patients (73%) had locally advanced disease (Stage III or IV) and 45 patients (85%) received a combination of radiotherapy and chemotherapy. No patients had distant metastases and all were alive and disease-free and had not received salvage surgery or chemotherapy within 6 months of completion of radiotherapy.

**Table 1:**
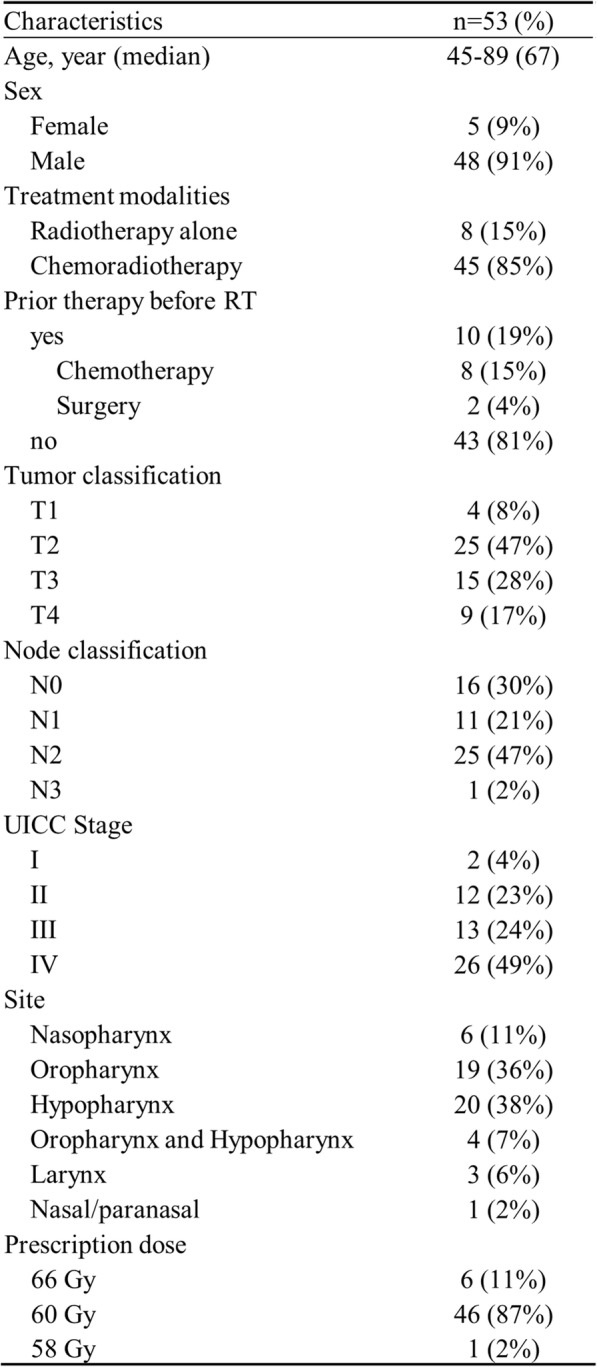
Patients Characteristics

### Organs at risk

Organs at risk (OARs) delineated in this study were as follows: spinal canal, spinal cord, brainstem, brain, eye, optic nerve, lens, optic chiasma, mandible, parotid gland, submandibular gland, oral cavity, sublingual gland, cochlea, thyroid, pharyngeal constrictor muscle, superior pharyngeal constrictor muscle (SPC), middle pharyngeal constrictor muscle (MPC), inferior pharyngeal constrictor muscle, temporomandibular joint, larynx, trachea, esophagus, temporal lobe, brachial plexus, internal auditory canal, tympanic cavity, eustachian tube, pituitary, lip, and planning target volume (PTV) 1 and PTV2. The guidelines reported by Sun et al. [14] were referred to for these OAR delineations. Regarding the parotid gland, the side that received the lower dose was selected for dosimetric analysis.

### Treatment

Radiotherapy was delivered using a tomotherapy system (Accuray®, Sunnyvale, CA, USA). All patients were immobilized with a face mask during their planning computed tomography acquisition of 1.25 mm slice thickness and all treatment sessions. The PTV1 was defined as the initial target volume and included the primary tumor, lymph nodes known to contain metastases, and bilateral neck node levels considered at risk of microscopic disease [7] with a 5-mm setup margin in all directions. The PTV2 was defined as the boost target volume and included the primary tumor and lymph nodes known to contain metastases with the same setup margin. PTV1 was treated with 40 Gy in 20 daily fractions and PTV2 with 60 or 66 Gy in 30 or 33 daily fractions with a two-step technique [9]. One patient stopped radiotherapy at 58 Gy in 29 fractions because of febrile neutropenia.

The highest priority was to deliver the prescription dose to > 95% of the PTVs. The maximum dosages of spinal canal planning organ at risk volume (PRV), brainstem PRV, and optic nerve PRV were restricted to 50 Gy, 54 Gy, and 50 Gy, respectively. The eyes, lens, parotid glands, submandibular glands, oral cavity, lips, mandible, larynx, and brain were spared as much as possible while delivering acceptable PTV coverage.

### HRQOL assessment

The European Organization for Research and Treatment of Cancer (EORTC) Quality of Life Questionnaire (QLQ-C30) and head and neck cancer module (QLQ-H&N35) were used for HRQOL assessments. The former includes a global health status scale, five functional scales, and nine symptom scales, and the latter comprises 18 symptom scales. The patients filled out the questionnaires before and 1, 3, 6, 9, and 12 months after completing radiotherapy for the first year, and at 6-month intervals for the following years. Patients who did not complete the baseline questionnaire or follow-up questionnaires for at least 6 months after radiotherapy were excluded. Questionnaires completed after disease relapse were excluded. According to the EORTC scoring procedure [6], all scales of the questionnaires were converted into scores that ranged from 0 to 100 points. A higher score for a global health status or a functional scale denotes a higher level of global health status or functioning, whereas a higher score on a symptom scale denotes more severe symptoms.

### Statistical analysis

Patients were divided into “severe-deterioration” and “mild-deterioration” groups on the basis of degree of deterioration from baseline for each follow-up time. Regarding QLQ-C30 scores, the severe-deterioration group was defined as patients who had ≥20 points deterioration [10], at least once, 6 or more months after completing treatment. Regarding QLQ-H&N35, the severe-deterioration group was defined as patients who had ≥40 points deterioration, at least once, 6 or more months after treatment. The remaining patients were allocated to a mild-deterioration group. Because 11 of the 18 symptom scales have only four possible variations in score, such as 0, 33, 67, or 100 points, 40 was selected as the threshold between the severe and mild groups.

Patient-related factors were categorized based on T stage (T1–2 vs. T3–4), N stage (N0–1 vs. N2–3), sex, age (≤67 vs. > 68), tumor location (nasopharynx or nasal cavity vs. other), and chemotherapy combination (with vs. without chemotherapy) [16]. These six categories and the mean dosage of the 34 OARs were compared between the severe- and mild-deterioration groups using Fisher’s exact test and Welch’s t-test. *P* value < 0.0013 was considered to denote statistical significance according to Bonferroni correction for 0.05 decision threshold. All statistical analyses were performed using R version 3.4.3 software (R Foundation, Vienna, Austria).

## Result

### Changes in HRQOL scores

**Fig. 2:**
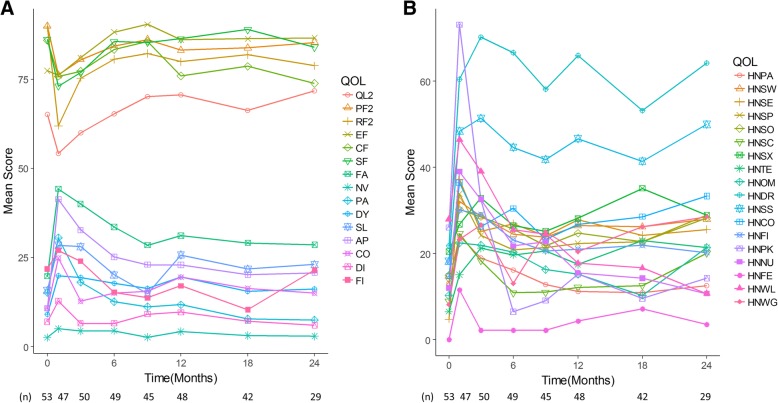
Chronological changes in health-related quality of life. Regarding global health status and functional scales (upper parts of 2A), higher score denotes better status. Regarding symptom scales (lower parts of 2A and all of 2B), higher score denotes worse status. Scores in many domains were worse 1 to 3 months after treatment; however, in two-thirds of the domains, they recovered to baseline levels by 6 to 9 months after treatment. Abbreviations: QL2 = global health status, PF2 = physical functioning, RF2 = role functioning, EF = emotional functioning, CF = cognitive functioning, SF = social functioning, FA = Fatigue, NV = Nausea and vomiting, PA = Pain, DY = Dyspnea, SL = Insomnia, AP = Appetite loss, CO = Constipation, DI = Diarrhea, FI = Financial difficulties, HNPA = Pain, HNSW = Swallowing, HNSE = Senses problems, HNSP = Speech problems, HNSO = Trouble with social eating, HNSC = Trouble with social contact, HNSX = Less sexuality, HNTE = Teeth, HNOM = Opening mouth, HNDR = Dry mouth, HNSS = Sticky saliva, HNCO = Coughing, HNFI = Felt ill, HNPK = Pain killers, HNNU = Nutritional supplements, HNFE = Feeding tube, HNWL = Weight loss, HNWG = Weight gain

The median duration of follow-up was 25.2 months (range, 7.1–44.3 months). Two patients (3.6%) were feeding tube-dependent 6 or more months after treatment. Figure 2 shows chronological changes in mean QOL scores for each domain. Generally, the scores were temporarily worse for 1 to 3 months after treatment, and recovered by 6 to 9 months after treatment. However, scores in one-third of the domains had not recovered to baseline levels 6 months after treatment, and remained at the same level thereafter. Domains with incomplete recovery included physical functioning, role functioning, fatigue, dyspnea, appetite loss, swallowing, sense problems, speech problems, trouble with social eating, dry mouth, sticky saliva, and coughing. The detailed findings are shown in the Additional file 1: Table S1.

### Risk factors for HRQOL outcomes

Table 2 shows comparisons of the mean of mean dosages to the MPC, SPC, and parotid gland between the severe- and mild-deterioration groups. Regarding trouble with social eating and coughing, the mean dosages to the SPC and parotid gland were significantly higher in the severe-deterioration than in the mild-deterioration group. Regarding nausea and vomiting, the mean dosage to the MPC was slightly higher in the severe deterioration than in the mild-deterioration group. No other patient- or treatment-related factors were associated with deterioration in any specific aspect of HRQOL (Additional file 2: Table S2).

**Table 2:**
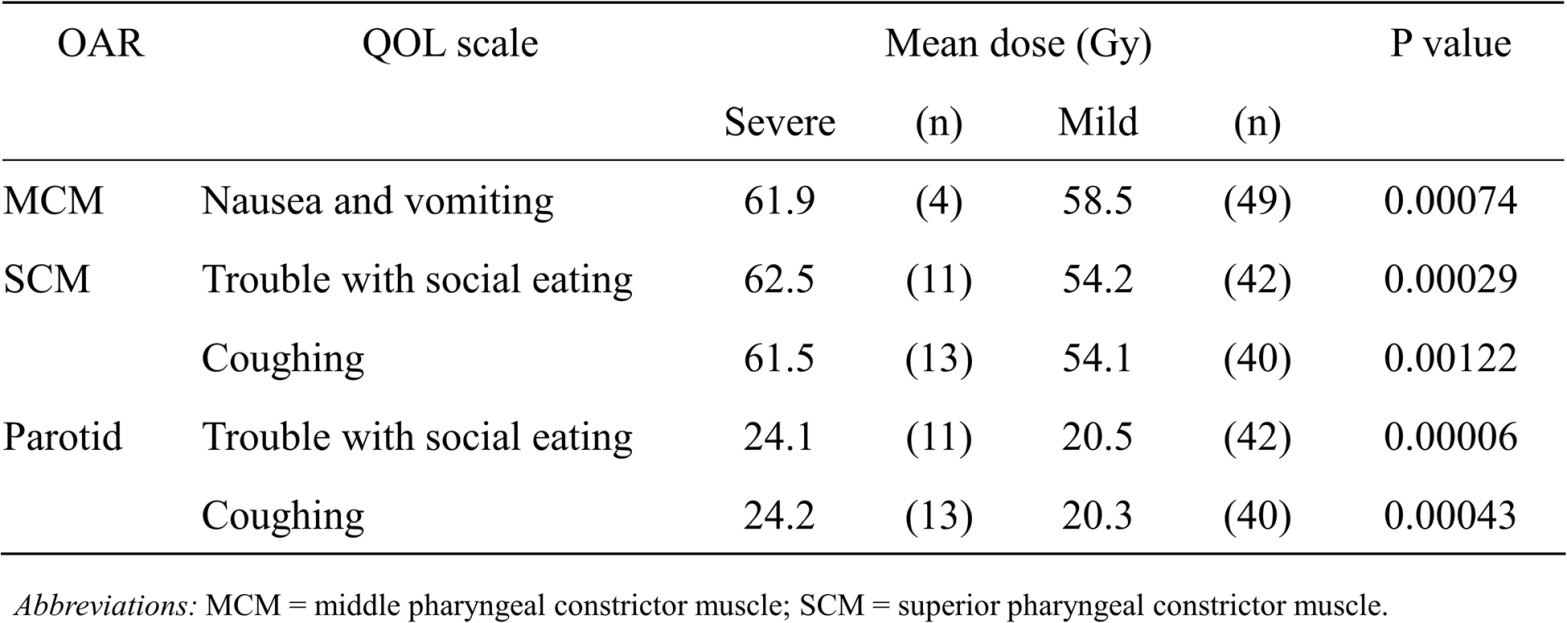
Mean dose of severe deterioration group versus mild deterioration group

## Discussion

Scores in two-thirds of the HRQOL domains recovered to pretreatment levels by 6 to 9 months after treatment, after having deteriorated temporarily. However, scores in one-third of the domains did not recover to baseline levels 6 or more months after treatment. We, therefore, used the time point of 6 months after treatment or later for assessing late-phase patient-reported symptoms.

Recently, Chera et al. reported a correlation between dosage to the SPC and patient-reported dysphagia on the basis of scores in the patient-reported outcome version of the Common Terminology Criteria for Adverse Events [4]. Our study confirmed that a higher dosage to the same OAR is a risk factor for patient-reported difficulty in social eating and coughing outcomes on the basis of EORTC H&N35 scores. Regarding feeding tube-dependency after treatment, several studies have reported that dosage to the SPC is a risk factor [3, 13, 17]. Because dysphagia and aspiration are the major reasons for feeding tube-dependence, our findings are compatible with those of previous studies.

Even though it is not intensively focused, it is not surprising that a higher dosage to the parotid gland causes not only xerostomia but also more severe difficulty with eating. In agreement with our study, Vainshtein et al. also reported that xerostomia contributes significantly to patient-reported dysphagia [15]. In addition, there is a clear relationship between xerostomia and dysphagia in patients with Sjogren syndrome [12]. Because all patients in our study were treated using IMRT, and the dosage to the parotid glands was kept as low as possible, dosage to the parotid gland was not significantly associated with xerostomia. Instead, the importance of the parotid gland for eating was extracted from our PRO data.

Regarding nausea/vomiting, our results suggest dosage to the MPC is a risk factor. However, when the two patients with feeding tubes were excluded from the analysis, the relationship between MPC dose and nausea/vomiting was no longer significant (data not shown). Therefore, feeding tube insertion may have contributed to nausea/vomiting. However, we hypothesize that vagus nerve activation [1] as a result of administering a high dose to the MPC may also have affected post-radiotherapy nausea/vomiting. The region of the MPC is densely innervated by a branch of vagus nerve, not only from a branch of the pharyngeal plexus cranially, but also from the recurrent laryngeal nerve caudally. Additionally, an MRI study has shown that the pharyngeal constrictor muscle is still inflamed and edematous 3 months after radiotherapy [11]. Therefore, we hypothesize that radiation-caused inflammation/edema in the MPC area can persist for a relatively long time, during which it can irritate the abundant branches of the vagus nerve, activating them and contributing to nausea and vomiting.

In our institution, we adopt 40 Gy as elective nodal irradiation (ENI) dosage for head and neck cancer patients; it is commonly used for other cancers (e.g. esophageal cancer and lung cancer). This dosage is lower than what is typically used in other institutions for head and neck cancer. The reasons why we adopt lower dosage for ENI are to reduce the risk of adverse events (e.g. dry mouth) by radiotherapy and to make salvage surgery easier in case of local relapse after definitive radiotherapy. We consider that lower ENI dosage is one of the important approaches to achieving better HRQOL outcomes.

Our study has several limitations that must be considered: 1) the retrospective manner of data collection, except for HRQOL scores, may have introduced some biases; 2) because several different physicians contoured the OARs, there may have been some variability in this; and 3) 60–66 Gy in 2 Gy fractions for definitive therapy is lower than the dose typically used in other international institutions.

## Conclusion

We found associations between mean dosages to the SPC and parotid gland and severe deterioration in social eating and coughing, and between mean dosage to the MPC and severe deterioration in nausea and vomiting 6 or more months after head and neck radiotherapy.

## Supplementary information

**Additional file 1: Table S1.** Chronological change of QOL scores.

**Additional file 2: Table S2.** Relationships between patient- or treatment-related factors and QOL score deterioration.

## Data Availability

The datasets generated and/or analyzed during the current study are not publicly available since the participants did not consent in sharing the data with third parties.

## Abbreviations

3DCRT: Three-dimensional conformal radiotherapy
HRQOL: Health-related quality of life
IMRT: Intensity modulated radiotherapy
QLQ-H&N35: European Organization for Research and Treatment of Cancer Quality of Life Questionnaire head and neck cancer module
MPC: Middle pharyngeal constrictor muscle
OARs: Organs at risk
PRO: Patient-reported outcomes
PRV: Planning organ at risk volume
PTV: Planning target volume
QLQ-C30: European Organization for Research and Treatment of Cancer Quality of Life Questionnaire
SPC: Superior pharyngeal constrictor muscle
