# Supplementary material for: Dosimetric factors associated with long-term patient-reported outcomes after definitive radiotherapy of patients with head and neck cancer
Source: Radiat Oncol. 2019 Dec 9;14:221. doi: 10.1186/s13014-019-1429-3 (PMC6902539; doi:10.1186/s13014-019-1429-3)
Supplement: Supplementary file 1 — Additional file 1: Table S1. Chronological change of QOL scores. [file 13014_2019_1429_MOESM1_ESM.zip › Table e1-1.pdf]

Table e1: Chronological change of QOL scores

QOL	baseline mean (SD)	1 month mean (SD)	1 month p value	3 month mean (SD)	3 month p value	6 month mean (SD)	6 month p value
QL2	65.1 (22.9)	54.2 (22.7)	0.002	60.0 (23.0)	0.184	65.2 (21.8)	0.925
PF2	89.8 (17.7)	76.2 (19.1)	<0.001	80.5 (18.1)	<0.001	84.3 (15.3)	<0.001
RF2	89.6 (21.2)	62.0 (28.7)	<0.001	75.3 (24.1)	<0.001	80.6 (23.7)	0.003
EF	77.4 (16.5)	76.0 (23.4)	0.795	80.1 (19.2)	0.110	88.2 (11.3)	0.001
CF	85.4 (15.5)	75.8 (19.2)	0.005	77.2 (19.4)	0.006	83.3 (16.9)	0.110
SF	86.2 (20.9)	73.0 (27.5)	0.003	76.8 (25.2)	0.029	85.5 (19.4)	0.851
FA	19.7 (18.3)	44.2 (21.6)	<0.001	40.0 (23.9)	<0.001	33.6 (21.4)	<0.001
NV	2.5 (8.9)	5.0 (10.9)	0.261	4.3 (10.0)	0.280	4.4 (13.5)	0.302
PA	15.1 (18.6)	30.5 (24.9)	0.001	18.0 (22.3)	0.562	12.6 (18.2)	0.358
DY	9.0 (16.3)	19.9 (22.7)	<0.001	19.3 (25.3)	0.001	17.7 (22.7)	0.004
SL	15.7 (25.0)	28.4 (31.1)	0.009	28.0 (31.1)	0.012	20.1 (23.6)	0.198
AP	10.7 (23.4)	41.3 (32.3)	<0.001	32.7 (29.7)	<0.001	25.2 (25.9)	0.004
CO	10.7 (20.4)	24.8 (20.2)	<0.001	12.7 (18.9)	0.607	15.0 (21.6)	0.359
DI	6.9 (17.7)	12.7 (18.0)	0.160	6.5 (13.4)	0.821	6.5 (13.4)	1.000
FI	21.8 (27.1)	27.0 (32.3)	0.562	23.9 (31.9)	0.402	15.2 (19.5)	0.077
HNPA	13.1 (16.0)	24.0 (19.9)	0.004	19.0 (18.0)	0.241	16.1 (17.2)	0.502
HNSW	14.7 (18.5)	32.2 (21.6)	0.001	28.6 (23.4)	0.024	24.5 (23.1)	0.038
HNSE	4.8 (10.6)	37.2 (27.7)	<0.001	25.2 (22.2)	<0.001	27.0 (19.5)	<0.001
HNSP	13.3 (19.2)	33.9 (24.0)	<0.001	24.2 (20.7)	0.068	20.9 (20.4)	0.066
HNSO	15.0 (18.0)	30.2 (23.2)	0.003	28.5 (18.6)	0.001	25.7 (20.2)	0.007
HNSC	9.0 (17.1)	24.6 (24.6)	<0.001	18.4 (14.6)	0.001	10.9 (14.3)	0.159
HNSX	20.5 (27.1)	26.8 (29.1)	0.109	32.9 (31.8)	0.006	26.5 (29.0)	0.271
HNTE	6.5 (18.9)	15.1 (22.3)	0.083	21.3 (27.3)	0.001	19.7 (26.2)	0.002
HNOM	10.3 (24.3)	22.5 (25.9)	0.044	22.0 (27.2)	0.028	20.3 (25.8)	0.086
HNDR	21.8 (20.8)	60.5 (24.4)	<0.001	70.2 (28.9)	<0.001	66.7 (25.6)	<0.001
HNSS	18.3 (26.1)	48.4 (28.7)	<0.001	51.4 (36.1)	<0.001	44.7 (28.9)	<0.001
HNCO	14.7 (20.3)	36.4 (23.9)	<0.001	26.2 (25.0)	0.018	30.5 (31.0)	0.002
HNFI	18.0 (26.3)	30.2 (31.1)	0.091	29.1 (28.3)	0.135	23.2 (25.2)	0.253
HNPK	26.0 (44.3)	73.2 (44.9)	<0.001	32.6 (47.4)	0.445	6.5 (25.0)	0.006
HNNU	12.0 (32.8)	39.0 (49.4)	0.010	32.6 (47.4)	0.006	21.7 (41.7)	0.058
HNFE	0.0 (0.0)	11.6 (32.4)	0.023	2.2 (14.7)	0.323	2.2 (14.9)	0.323
HNWL	28.0 (45.4)	46.5 (50.5)	0.016	39.1 (49.3)	0.323	25.5 (44.1)	0.660
HNWG	8.2 (27.7)	23.2 (42.7)	0.083	26.7 (44.7)	0.006	13.0 (34.1)	0.533

Baseline and each follow-up point score were compared by paired-t test.
