# Supplementary material for: Dosimetric factors associated with long-term patient-reported outcomes after definitive radiotherapy of patients with head and neck cancer
Source: Radiat Oncol. 2019 Dec 9;14:221. doi: 10.1186/s13014-019-1429-3 (PMC6902539; doi:10.1186/s13014-019-1429-3)
Supplement: Supplementary file 1 — Additional file 1: Table S1. Chronological change of QOL scores. [file 13014_2019_1429_MOESM1_ESM.zip › Table e1-2.pdf]

9 month		12 month		18 month		24 month	
mean (SD)	p value	mean (SD)	p value	mean (SD)	p value	mean (SD)	p value
70.1 (23.2)	0.330	70.6 (19.3)	0.060	66.3 (23.1)	0.948	71.7 (22.8)	0.380
86.1 (15.1)	0.004	83.1 (18.9)	<0.001	83.8 (18.2)	<0.001	85.3 (21.0)	0.018
82.2 (22.3)	0.005	79.9 (24.3)	0.003	81.8 (25.7)	0.018	78.7 (29.8)	0.019
90.3 (11.5)	<0.001	86.0 (12.4)	0.010	86.3 (14.2)	0.014	86.6 (15.9)	0.174
85.6 (19.2)	0.467	75.9 (20.0)	0.001	78.6 (21.6)	0.020	73.8 (26.2)	0.004
85.2 (21.9)	0.724	86.3 (18.2)	0.843	88.9 (17.9)	0.621	83.9 (24.6)	0.631
28.4 (21.8)	0.040	31.3 (23.2)	0.004	29.1 (20.9)	0.005	28.5 (22.8)	0.007
2.6 (8.7)	1.000	4.2 (9.4)	0.351	3.1 (9.1)	0.643	2.9 (10.0)	0.375
11.1 (17.0)	0.459	11.7 (15.9)	0.419	7.8 (13.2)	0.058	7.5 (12.3)	0.364
16.3 (18.3)	0.001	19.4 (22.6)	0.002	15.5 (21.0)	0.027	16.1 (21.1)	0.050
14.8 (20.8)	0.700	25.7 (25.9)	0.008	21.7 (22.9)	0.141	23.0 (28.3)	0.083
23.0 (25.4)	0.005	22.9 (28.5)	0.002	20.2 (24.3)	0.005	20.7 (25.8)	0.036
15.6 (18.3)	0.160	19.4 (26.5)	0.044	16.3 (19.7)	0.164	14.9 (16.9)	0.009
9.1 (15.0)	0.323	9.6 (16.9)	0.473	7.1 (13.8)	0.643	6.0 (15.9)	0.537
13.6 (23.1)	0.105	17.0 (22.0)	0.193	10.3 (18.8)	0.029	21.4 (30.4)	0.879
12.8 (17.8)	0.917	11.2 (16.4)	0.645	10.9 (13.8)	0.285	12.6 (16.4)	0.620
23.8 (23.9)	0.103	26.4 (25.3)	0.017	26.2 (23.7)	0.018	28.0 (27.9)	0.007
24.0 (20.0)	<0.001	27.9 (28.8)	<0.001	24.2 (21.2)	<0.001	25.6 (26.2)	<0.001
21.5 (21.7)	0.103	22.3 (26.4)	0.282	22.8 (25.7)	0.026	29.0 (28.1)	0.004
21.8 (16.9)	0.067	24.7 (23.3)	0.049	22.8 (20.0)	0.011	28.3 (24.2)	0.003
11.0 (13.0)	0.291	12.1 (19.3)	0.304	12.5 (15.2)	0.010	20.1 (26.2)	0.008
25.2 (27.8)	0.127	28.2 (28.4)	0.060	35.1 (27.7)	<0.001	29.0 (30.7)	0.162
20.6 (25.5)	<0.001	17.5 (24.7)	0.003	23.0 (29.0)	<0.001	21.4 (27.5)	0.009
16.3 (25.6)	0.336	15.2 (26.0)	0.452	10.3 (22.7)	0.584	21.4 (31.7)	0.030
58.1 (27.3)	<0.001	65.9 (31.8)	<0.001	53.2 (28.6)	<0.001	64.3 (31.3)	<0.001
41.9 (27.3)	<0.001	46.7 (32.1)	<0.001	41.5 (27.7)	<0.001	50.0 (35.7)	0.001
23.3 (27.7)	0.040	26.8 (27.8)	0.006	28.6 (27.1)	<0.001	33.3 (30.1)	<0.001
20.6 (26.5)	0.361	21.0 (22.6)	0.349	22.0 (21.9)	0.198	20.2 (24.6)	0.202
9.1 (29.1)	0.002	15.2 (36.3)	0.096	9.5 (29.7)	0.018	14.3 (35.6)	0.103
22.7 (42.4)	0.058	15.6 (36.7)	0.183	14.3 (35.4)	0.711	10.7 (31.5)	0.327
2.3 (15.1)	0.323	4.3 (20.6)	0.160	7.1 (26.1)	0.083	3.6 (18.9)	0.327
24.4 (43.5)	0.372	17.8 (38.7)	0.133	16.7 (37.7)	0.291	10.7 (31.5)	0.265
25.0 (43.8)	0.033	20.5 (40.8)	0.323	26.1 (44.5)	0.031	28.6 (46.0)	0.212
