# Supplementary material for: Dosimetric factors associated with long-term patient-reported outcomes after definitive radiotherapy of patients with head and neck cancer
Source: Radiat Oncol. 2019 Dec 9;14:221. doi: 10.1186/s13014-019-1429-3 (PMC6902539; doi:10.1186/s13014-019-1429-3)
Supplement: Supplementary file 2 — Additional file 2: Table S2. Relationships between patient- or treatment-related factors and QOL score deterioration. [file 13014_2019_1429_MOESM2_ESM.zip › Table e2-1.pdf]

Table e2: Patient- or treatment-related factors

(n)		Age			T stage			N stage		
QOL	group	≦ 67	＞ 68	p value	T 1-2	T 3-4	p value	N 0-1	N 2-3	p value
QL2	severe	7	7	1.00	7	7	0.76	7	7	1.00
	mild	20	19		22	17		20	19	
PF2	severe	9	9	1.00	8	10	0.38	10	8	0.77
	mild	18	17		21	14		17	18	
RF2	severe	12	10	0.78	12	10	1.00	11	11	1.00
	mild	15	16		17	14		16	15	
EF	severe	4	4	1.00	3	5	0.44	3	5	0.47
	mild	23	22		26	19		24	21	
CF	severe	9	6	0.54	9	6	0.76	8	7	1.00
	mild	18	20		20	18		19	19	
SF	severe	7	5	0.74	6	6	0.75	6	6	1.00
	mild	20	21		23	18		21	20	
FA	severe	15	14	1.00	17	12	0.59	14	15	0.78
	mild	12	12		12	12		13	11	
NV	severe	0	4	0.05	1	3	0.32	2	2	1.00
	mild	27	22		28	21		25	24	
PA	severe	3	4	0.70	5	2	0.44	3	4	0.70
	mild	24	22		24	22		24	22	
DY	severe	13	11	0.78	13	11	1.00	12	12	1.00
	mild	14	15		16	13		15	14	
SL	severe	16	10	0.17	16	10	0.41	15	11	0.41
	mild	11	16		13	14		12	15	
AP	severe	20	13	0.09	16	17	0.27	14	19	0.16
	mild	7	13		13	7		13	7	
CO	severe	11	14	0.41	14	11	1.00	11	14	0.41
	mild	16	12		15	13		16	12	
DI	severe	8	8	1.00	5	11	0.04	9	7	0.77
	mild	19	18		24	13		18	19	
FI	severe	10	4	0.12	8	6	1.00	8	6	0.76
	mild	17	22		21	18		19	20	
