# Supplementary material for: Dosimetric factors associated with long-term patient-reported outcomes after definitive radiotherapy of patients with head and neck cancer
Source: Radiat Oncol. 2019 Dec 9;14:221. doi: 10.1186/s13014-019-1429-3 (PMC6902539; doi:10.1186/s13014-019-1429-3)
Supplement: Supplementary file 2 — Additional file 2: Table S2. Relationships between patient- or treatment-related factors and QOL score deterioration. [file 13014_2019_1429_MOESM2_ESM.zip › Table e2-2.pdf]

HNPA	severe	3	1	0.61	3	1	0.62	1	3	0.61
	mild	24	25		26	23		26	23	
HNSW	severe	7	4	0.50	6	5	1.00	5	6	0.74
	mild	20	22		23	19		22	20	
HNSE	severe	13	7	0.16	10	10	0.78	7	13	0.09
	mild	14	19		19	14		20	13	
HNSP	severe	4	4	1.00	3	5	0.44	5	3	0.70
	mild	23	22		26	19		22	23	
HNSO	severe	7	4	0.50	4	7	0.19	5	6	0.74
	mild	20	22		25	17		22	20	
HNSC	severe	3	3	1.00	1	5	0.08	5	1	0.19
	mild	24	23		28	19		22	25	
HNSX	severe	5	5	1.00	3	7	0.16	5	5	1.00
	mild	22	21		26	17		22	21	
HNTE	severe	10	3	0.05	9	4	0.34	4	9	0.12
	mild	17	23		20	20		23	17	
HNOM	severe	3	4	0.70	3	4	0.69	3	4	0.70
	mild	24	22		26	20		24	22	
HNDR	severe	17	12	0.27	14	15	0.41	16	13	0.59
	mild	10	14		15	9		11	13	
HNSS	severe	16	8	0.05	12	12	0.59	14	10	0.41
	mild	11	18		17	12		13	16	
HNCO	severe	8	5	0.53	5	8	0.21	6	7	0.76
	mild	19	21		24	16		21	19	
HNFI	severe	5	2	0.42	4	3	1.00	2	5	0.25
	mild	22	24		25	21		25	21	
HNPK	severe	2	3	0.67	3	2	1.00	3	2	1.00
	mild	25	23		26	22		24	24	
HNNU	severe	7	6	1.00	5	8	0.21	8	5	0.53
	mild	20	20		24	16		19	21	
HNFE	severe	1	3	0.35	1	3	0.32	1	3	0.35
	mild	26	23		28	21		26	23	
HNWL	severe	9	8	1.00	8	9	0.56	5	12	0.04
	mild	18	18		21	15		22	14	
HNWG	severe	16	10	0.17	17	9	0.17	16	10	0.17
	mild	11	16		12	15		11	16	
