# Supplementary material for: Dosimetric factors associated with long-term patient-reported outcomes after definitive radiotherapy of patients with head and neck cancer
Source: Radiat Oncol. 2019 Dec 9;14:221. doi: 10.1186/s13014-019-1429-3 (PMC6902539; doi:10.1186/s13014-019-1429-3)
Supplement: Supplementary file 2 — Additional file 2: Table S2. Relationships between patient- or treatment-related factors and QOL score deterioration. [file 13014_2019_1429_MOESM2_ESM.zip › Table e2-3.pdf]

(n)		Sex			Site			chemotherapy		
QOL	group	female	male	p value	nasal / paranasal	the other	p value	yes	no	p value
QL2	severe	2	12	0.60	2	12	1.00	10	4	0.19
	mild	3	36		5	34		35	4	
PF2	severe	3	15	0.32	3	15	0.68	16	2	0.70
	mild	2	33		4	31		29	6	
RF2	severe	3	19	0.64	5	17	0.11	19	3	1.00
	mild	2	29		2	29		26	5	
EF	severe	1	7	0.57	2	6	0.28	8	0	0.33
	mild	4	41		5	40		37	8	
CF	severe	2	13	0.61	3	12	0.39	12	3	0.67
	mild	3	35		4	34		33	5	
SF	severe	2	10	0.32	3	9	0.18	11	1	0.67
	mild	3	38		4	37		34	7	
FA	severe	3	26	1.00	5	24	0.44	26	3	0.44
	mild	2	22		2	22		19	5	
NV	severe	0	4	1.00	0	4	1.00	3	1	0.49
	mild	5	44		7	42		42	7	
PA	severe	0	7	1.00	2	5	0.23	6	1	1.00
	mild	5	41		5	41		39	7	
DY	severe	3	21	0.65	3	21	1.00	23	1	0.06
	mild	2	27		4	25		22	7	
SL	severe	3	23	0.67	6	20	0.05	22	4	1.00
	mild	2	25		1	26		23	4	
AP	severe	5	28	0.14	6	27	0.23	29	4	0.46
	mild	0	20		1	19		16	4	
CO	severe	4	21	0.18	5	20	0.23	23	2	0.26
	mild	1	27		2	26		22	6	
DI	severe	2	14	0.63	2	14	1.00	14	2	1.00
	mild	3	34		5	32		31	6	
FI	severe	3	11	0.11	2	12	1.00	14	0	0.09
	mild	2	37		5	34		31	8	
