# Supplementary material for: Dosimetric factors associated with long-term patient-reported outcomes after definitive radiotherapy of patients with head and neck cancer
Source: Radiat Oncol. 2019 Dec 9;14:221. doi: 10.1186/s13014-019-1429-3 (PMC6902539; doi:10.1186/s13014-019-1429-3)
Supplement: Supplementary file 2 — Additional file 2: Table S2. Relationships between patient- or treatment-related factors and QOL score deterioration. [file 13014_2019_1429_MOESM2_ESM.zip › Table e2-4.pdf]

HNPA	severe	0	4	1.00	2	2	0.08	4	0	1.00
	mild	5	44		5	44		41	8	
HNSW	severe	2	9	0.27	2	9	0.63	10	1	1.00
	mild	3	39		5	37		35	7	
HNSE	severe	3	17	0.35	4	16	0.40	19	1	0.23
	mild	2	31		3	30		26	7	
HNSP	severe	2	6	0.16	3	5	0.06	7	1	1.00
	mild	3	42		4	41		38	7	
HNSO	severe	1	10	1.00	3	8	0.15	10	1	1.00
	mild	4	38		4	38		35	7	
HNSC	severe	0	6	1.00	2	4	0.17	6	0	0.57
	mild	5	42		5	42		39	8	
HNSX	severe	0	10	0.57	3	7	0.11	10	0	0.33
	mild	5	38		4	39		35	8	
HNTE	severe	2	11	0.59	2	11	1.00	13	0	0.18
	mild	3	37		5	35		32	8	
HNOM	severe	0	7	1.00	3	4	0.04	7	0	0.58
	mild	5	41		4	42		38	8	
HNDR	severe	4	25	0.36	5	24	0.44	25	4	1.00
	mild	1	23		2	22		20	4	
HNSS	severe	3	21	0.65	4	20	0.69	22	2	0.27
	mild	2	27		3	26		23	6	
HNCO	severe	2	11	0.59	3	10	0.34	12	1	0.66
	mild	3	37		4	16		33	7	
HNFI	severe	2	5	0.12	2	5	0.23	6	1	1.00
	mild	3	43		5	41		39	7	
HNPK	severe	0	5	1.00	2	3	0.12	3	2	0.16
	mild	5	43		5	43		42	6	
HNNU	severe	1	12	1.00	1	12	0.67	12	1	0.66
	mild	4	36		6	34		33	7	
HNFE	severe	0	4	1.00	1	3	0.44	4	0	1.00
	mild	5	44		6	43		41	8	
HNWL	severe	2	15	0.65	6	11	0.003	15	2	1.00
	mild	3	33		1	35		30	6	
HNWG	severe	1	25	0.35	5	21	0.25	21	5	0.47
	mild	4	23		2	25		24	3	
